# The histone lysine acetyltransferase KAT2B inhibits cholangiocarcinoma growth: evidence for interaction with SP1 to regulate NF2-YAP signaling

**DOI:** 10.1186/s13046-024-03036-5

**Published:** 2024-04-19

**Authors:** Wenbo Ma, Jinqiang Zhang, Weina Chen, Nianli Liu, Tong Wu

**Affiliations:** Department of Pathology and Laboratory Medicine, Tulane University School of Medicine, 1430 Tulane Avenue, SL-79, New Orleans, LA 70112 USA

**Keywords:** KAT2B, Histone acetyltransferase, Cholangiocarcinoma, NF2, YAP

## Abstract

**Background:**

Cholangiocarcinoma (CCA) is a highly malignant cancer of the biliary tract with poor prognosis. Further mechanistic insights into the molecular mechanisms of CCA are needed to develop more effective target therapy.

**Methods:**

The expression of the histone lysine acetyltransferase KAT2B in human CCA was analyzed in human CCA tissues. CCA xenograft was developed by inoculation of human CCA cells with or without KAT2B overexpression into SCID mice. Western blotting, ChIP-qPCR, qRT-PCR, protein immunoprecipitation, GST pull-down and RNA-seq were performed to delineate KAT2B mechanisms of action in CCA.

**Results:**

We identified KAT2B as a frequently downregulated histone acetyltransferase in human CCA. Downregulation of KAT2B was significantly associated with CCA disease progression and poor prognosis of CCA patients. The reduction of KAT2B expression in human CCA was attributed to gene copy number loss. In experimental systems, we demonstrated that overexpression of KAT2B suppressed CCA cell proliferation and colony formation in vitro and inhibits CCA growth in mice. Mechanistically, forced overexpression of KAT2B enhanced the expression of the tumor suppressor gene NF2, which is independent of its histone acetyltransferase activity. We showed that KAT2B was recruited to the promoter region of the NF2 gene via interaction with the transcription factor SP1, which led to enhanced transcription of the NF2 gene. KAT2B-induced NF2 resulted in subsequent inhibition of YAP activity, as reflected by reduced nuclear accumulation of oncogenic YAP and inhibition of YAP downstream genes. Depletion of NF2 was able to reverse KAT2B-induced reduction of nuclear YAP and subvert KAT2B-induced inhibition of CCA cell growth.

**Conclusions:**

This study provides the first evidence for an important tumor inhibitory effect of KAT2B in CCA through regulation of NF2-YAP signaling and suggests that this signaling cascade may be therapeutically targeted for CCA treatment.

**Supplementary Information:**

The online version contains supplementary material available at 10.1186/s13046-024-03036-5.

## Introduction

Cholangiocarcinoma (CCA) is a highly malignant cancer of the biliary system with poor prognosis [1–3]. The incidence of CCA has been increasing over the past 20 years and the mortality of the disease remains unacceptably high. While surgical resection is applicable to early stage of the disease, most CCA patients are diagnosed at an advanced stage which precludes curative surgical intervention [4, 5]. Therefore, further mechanistic insights into the pathogenesis of CCA are needed to identify effective therapeutic targets.

In recent years, aberrant epigenetic alterations have been recognized as important events in cancer development and progression. Epigenetic regulation refers to reversible modifications on DNA and histones, which plays critical roles in chromatin remodeling and gene transcription without affecting genome sequence [6–8]. In contrast to the irreversibility of genetic changes, the reversibility of epigenetic modifications has made epigenetic machinery an attractive target for drug development [9]. A number of small-molecule inhibitors have been developed against epigenetic enzymes and some of them have showed impressive effects in anti-cancer therapy [10, 11], including the treatment of CCA in pre-clinical studies [12]. In this context, previous studies from our group have documented the roles of two histone methyltransferases (HMTs), EZH2 (enhancer of Zeste 2) and G9a/EHMT2 (euchromatic histone-lysine N-methyltransferase 2), in CCA [13–15]. However, the role of histone acetyltransferases (HATs) in CCA remains to be further defined.

Histone acetyltransferases (HATs) refer to a group of enzymes that catalyze the transfer of an acetyl group from acetyl CoA to form ε-N-acetyl lysine in histone proteins; this process is well known to play important roles in chromatin remodeling and gene expression [16, 17]. While aberrant expression and activity of HATs have been observed in various human cancers [16, 18], the potential role of HATs in CCA is largely unknown.

The current study was designed to investigate the effect and mechanism of the histone acetyltransferase KAT2B (Lysine acetyltransferase 2B) in human CCA. We herein report that KAT2B is a frequently downregulated histone acetyltransferase in human CCA tissues and that KAT2B downregulation is associated with CCA disease progression and poor prognosis of CCA patients. We show that KAT2B suppresses CCA cell proliferation and colony formation in vitro and inhibits CCA growth in mice. Our findings indicate that KAT2B inhibits CCA cell growth through interaction with the transcription factor SP1 to induce the expression of the tumor suppressor NF2 which inhibits oncogenic YAP signaling. This study provides the first evidence for an important tumor inhibitory effect of KAT2B in CCA.

## Materials and methods

### Materials

Dulbecco’s modified minimum essential medium (DMEM) and fetal bovine serum (FBS) were purchased from Sigma (St. Louis, MO). RT-qPCR, ChIP-qPCR primers were synthesized at Thermo Fisher Scientific (Waltham, MA) and are listed in Supplementary Table 1 and Supplementary Table 2. The pMSCVpuro-KAT2B vector (#63,705), pMSCVpuro empty control vector (#12,570), pGEX-GST-SP1 (#27,264) and pGEX-GST (#129,572) were purchased from Addgeene (Cambridge, MA). The siRNA duplexes were synthesized at Integrated DNA Technologies (Coraville, IA). The sequences of siRNAs are listed in Supplementary Table 3. Lipofectamine™ 3000 reagent, glutamine and antibiotics were obtained from Invitrogen (Carlsbad, CA). RIPA Lysis Buffer (#89,901), BL21(DE3) Competent Cells (#EC0114), Subcellular Protein Fractionation Kit (#78,840), Pierce™ GST Protein Interaction Pull-Down Kit (#21,516) and Isopropyl β-D-1-thiogalactopyranoside (IPTG) Solution (#R1171) were purchased from Thermo Fisher Scientific (Waltham, MA). The EpiQuik Nuclear Extraction Kit (#OP-0002-1) was purchased from Epigentek (Farmingdale, NY). Recombinant KAT2B-Flag protein was purchased from Active Motif (Carlsbad, CA). Rabbit polyclonal antibodies against KAT2B, GST, YAP and NF2 were purchased from Proteintech (Rosemont, IL). Rabbit polyclonal antibody against NFYB was purchased from GeneTex (Irvine, CA). Rabbit monoclonal antibodies against KAT2B, YY1, Flag and mouse monoclonal antibodies against Ki67/α-Tubulin were purchased from Cell Signaling Technology (Danvers, MA). Rabbit monoclonal antibody against SP1 and Rabbit polyclonal antibodies against NFYA/NFYC were purchased from ABclonal (Woburn, MA). Mouse monoclonal antibody against β-actin was purchased from Sigma (St. Louis, MO). IRDye goat anti-mouse/rabbit IgG secondary antibodies were purchased from LI-COR Biosciences (Lincoln, NE).

### Cell culture and transfections

Human CCA cell lines (SG231, HuCCT1) were cultured in Dulbecco’s modified Eagle’s medium (Invitrogen, Carlsbad, CA) containing 10% fetal bovine serum (Sigma-Aldrich) and antibiotics (100 U/mL penicillin and 100 µg/mL streptomycin) in a humidified 5% CO2 incubator at 37 °C.

For transfections, NF2 and SP1 siRNAs and their respective scramble controls were transfected into CCA cells using Lipofectamine™ 3000 reagent. Following transfections, the cells were analyzed for proliferation and other parameters as described in the manuscript.

For establishment of CCA cells with stable overexpression of KAT2B, HuCCT1 and SG231 cells were transfected with pMSCV-KAT2B vector or empty control vector. After 48 h of transfection, the cells were cultured in DMEM medium containing 1 µg/ml Puromycin (Calbiochem, San Diego, CA). The selection medium was replaced every 3 days for the next 4 weeks. Subsequently, distinct colonies of surviving cells were transferred onto 6-well plates and the cultures were maintained under the same selection medium. Following transfections, the cells were analyzed for proliferation, invasion, and specific protein levels.

### Cell proliferation WST-1 assay

Cell proliferation WST-1 assay was performed according to the manufacturer’s instruction. For cells with overexpression of KAT2B, 2 × 10^3^ cells were seeded onto each well of 96-well plates and cultured for 5 days. For NF2 siRNA transfection, 2 × 10^3^ cells with stable overexpression of KAT2B were seeded onto each well of 96-well plates and the cells were transfected with siRNAs in the presence of Lipofectamine™ 2000 reagent for 4 h; the cells were then continued cultured in fresh DMEM medium for additional 5 days. To determine cell proliferation, 10 µl WST-1 reagent was added to each well and the cells were incubated for 1 h at 37 °C and 5% CO_2_. A450 nm was measured using an automatic ELISA plate reader.

### Clonogenicity assay

For anchorage-dependent colony formation assay, CCA cells transfected with or without KAT2B overexpression were seeded onto conventional 6-well plates (500 cells/ well). After culture for 14 days, the cells were fixed with methanol and stained with 0.1% crystal violet. For anchorage-independent colony formation assay, cells were cultured in ultra-low attachment plates (Corning, NY) as previously described [19–21]. 1 × 10^3^ SG231cells/well or 2 × 10^3^ HuCCT1 cells/well were seeded onto 24-well ultra-low attachment plates in DMEM medium containing 1% FBS (for SG231 cells) or 5% FBS (for HuCCT1 cells). Following culture for 10 days (for SG231) or 14 days (for HuCCT1), the cell spheroids were imaged by using an inverted Olympus microscope.

### Protein extraction and Western blotting

For whole cell protein extraction, the cells were washed twice with ice-cold PBS and lysed in RIPA buffer. After sonication on ice, the cell lysates were centrifuged at 13,000 rpm for 10 min at 4 °C and the supernatants were collected for Western blotting. For nuclear protein extraction, we followed the manufacture’s instruction using EpiQuik Nuclear Extraction Kit. Briefly, the cells were scraped with 1× buffer NE1 (plus Protease Inhibitor Cocktail (PIC)) and left on ice for 10 min and centrifuged for 10 min at 12,000 rpm (4 °C). The pellets were then washed with ice-cold PBS and resuspended in 1× buffer NE2 (containing DTT and PIC). The samples were then homogenized and left on ice for 15 min with vortex (5 s) every 3 min. After centrifugation at 14,000 rpm for 10 min, the supernatants were collected as nuclear proteins. The insoluble chromatin fraction was extracted according to the manufacturer’s instructions provided by Subcellular Protein Fractionation Kit (Thermo Fisher Scientific, Waltham, MA). The protein concentrations were measured using the Bio-Rad Protein Assay Kit (Bio-Rad, Hercules, CA).

For Western blotting analysis, samples were boiled for 5 min in protein loading buffer with 2-mercaptoethanol and subjected to 10% sodium dodecyl sulfate polyacrylamide gel electrophoresis (SDS-PAGE). The proteins were then transferred onto the nitrocellulose membrane (BioRad). Non-specific binding was blocked by incubating the membranes in PBST (0.1% Tween 20 in PBS) containing 5% nonfat milk for 1 h at room temperature. The membranes were then incubated overnight at 4 °C with individual primary antibodies at the dilutions recommended by the manufacturers in PBST containing 5% nonfat milk. Following four washes with PBST, the membranes were incubated with the IRDye secondary antibodies at 1: 5000 dilutions in PBST containing 5% nonfat milk for 1 h at room temperature. After four washes with PBST, the ODYSSEY Infrared Imaging System (Licor, Lincoln, NE) was used to visualize protein bands.

### Real-time quantitative PCR (qRT-PCR)

Total RNA was extracted using Tri-zol Reagents (Invitrogen, Carlsbad, CA) following the manufacturer’s instructions. Reverse transcription was performed with iScript Supermix (Bio-Rad, Hercules, CA). Quantitative PCR was performed with the Bio-Rad SYBR® Green Supermix in a C1000 thermal cycler (Bio-Rad). The PCR conditions were 15 min at 95 °C, followed by 35 cycles of 15s at 94 °C, 30s at 55 °C and 30s at 72 °C, and finally 10 min at 72 °C. The PCR primer sequences are listed in Supplementary Table 1. β-actin was used as the internal control. Results were analyzed by using CFX Manager Software version 3.1 (Bio-Rad). The expression level of mRNA was normalized to the internal control gene and relative change was calculated by using the 2^−ΔΔCT^ method.

### Chromatin immunoprecipitation (ChIP)

Cells were cross-linked by 1% formaldehyde for 10 min. Chromosome DNA was extracted according to the manufacturer’s instructions provided by SimpleChIP ® Plus Enzymatic Chromatin IP Kit (Cell signaling, Danvers, MA) and precipitated by using specific anti-KAT2B rabbit antibody or anti-SP1 rabbit antibody. Rabbit IgG was used as negative control. The PCR conditions were 15 min at 95 °C, followed by 35 cycles of 15s at 94 °C, 30s at 55 °C and 30s at 72 °C, and then 10 min at 72 °C. The ChIP-PCR primer sequences are listed in Supplementary Table 2.

### Protein immunoprecipitation

CCA cells (1 × 10^7^) were lysed in 1 mL Pierce IP Lysis Buffer (Thermo Fisher Scientific) containing phosphatase and protease inhibitors. Then, 500 µl cell lysates were used for immunoprecipitation with specific antibodies. In brief, cell lysate were incubated with 10 µg antibodies by rotation at 4 °C overnight and then with the addition of 50 µL PureProteome Protein A/G Mix Magnetic Beads (MilliporeSigma #LSKMAGAG10) at 4 °C for 4 more hours. The samples were collected by magnetic stand, followed by washing five times with a beads wash solution (50 mmol/L Tris-HCl [pH 7.6], 150 mmol/L NaCl, 1 mmol/L EDTA, and 0.1% NP-40) and washing three times with phosphate-buffered saline. After boiling for 5 min in protein loading buffer, the samples were used for Western blotting analysis with specific antibodies.

### GST pull-down assay

To produce glutathione S-transferase (GST) and GST-SP1 fusion protein, pGEX-GST control vector and pGEX-GST-SP1 were transfected into BL21 (DE3) competent cells respectively. Protein expression was induced by 0.5mM ispropylb-D-1-thiogalactopyranoside (IPTG). GST pull-down assays were performed according to the manufacturer’s protocol provided by Pierce™ GST Protein Interaction Pull-Down Kit (Thermo Scientific, Waltham, MA). Briefly, the recombinant GST-SP1 or GST protein was incubated with Glutathione Agarose at 4 °C for 2 h. To minimize the possibility of nonspecific binding, we increased the washing process to 8 times using the wash buffer recommended by the manufacturer’s instructions (1:1 wash solution of TBS: Pull-Down Lysis Buffer). After washing eight times, the Glutathione Agarose was incubated with FLAG-tagged KAT2B recombinant protein (Active Motif, Carlsbad, CA) at 4 °C for 2 h, followed by washing for additional eight times with the above-indicated wash buffer. The samples were then analyzed by Western blotting.

### Immunohistochemistry (IHC)

The CCA tumor tissues were fixed in 10% buffered formalin and embedded in paraffin. Sections of 4 μm thickness were deparaffinized and processed for hematoxylin and eosin (H&E) staining and immunohistochemistry. Primary antibodies against KAT2B, YAP, NF2, or Ki67 were diluted in 1× PBS containing 4% horse serum, 0.4 mg/ml methiolate and 0.2% Triton-X100. After blocking with Peroxidazed 1 (Biocare Medical, Pike Lane Concord, CA) for 5 min, the slides were incubated with primary antibodies at room temperature for 1 h. The slides were then washed with TBS and incubated with horseradish peroxidase-conjugated second antibody at room temperature for 1 h. After washing with TBS, the slides were incubated for 5 min at room temperature with 3,3′-diaminobenzidine (DAB) for chromogenic development.

### RNA-sequencing (RNA‐seq)

Total RNA was extracted from HuCCT1 cells using Trizol reagent (Invitrogen, CA, USA) following the manufacturer’s procedure. Library preparation, high-throughput sequencing and data analysis were done by LC Sciences (Houston, TX). Briefly, RNA sequencing library was prepared following Illumina’s TruSeq-stranded-mRNA sample preparation protocol. RNA integrity was checked with Agilent Technologies 2100 Bioanalyzer. Poly(A) tail-containing mRNAs were purified using oligo-(dT) magnetic beads with two rounds of purification. After purification, poly(A) RNA was fragmented using divalent cation buffer in elevated temperature, followed by DNA library construction. Quality control analysis and quantification of the sequencing library were performed using Agilent Technologies 2100 Bioanalyzer High Sensitivity DNA Chip. Paired-ended sequencing was performed on Illumina’s NovaSeq 6000 sequencing system.

### Bioinformatics analyses

The gene expression profiles of human CCA and non-cancerous liver tissues were downloaded from the GEO datasets (GSE26566, GSE107943, GSE76297, GSE119336) and TCGA (The Cancer Genome Atlas) database. GSE26566 consisted of 104 CCA samples and 59 non-tumor liver samples. GSE107943 consisted of 30 CCA samples and 27 non-CCA samples. GSE76297 consisted of 90 CCA samples and 90 paired non-CCA samples. GSE119336 consisted of 15 CCA samples and 15 paired non-CCA samples. TCGA database consisted of 36 CCA samples and 9 non-CCA samples. Analysis of the TCGA-CHOL survival data was performed by using the online tool GEPIA (http://gepia.cancer-pku.cn/detail.php) with a standard processing pipeline [22]. The correlation between KAT2B expression and the overall survival of CCA patients was further assessed by using GSE244807 dataset.

To compare KAT2B expression levels between CCA and bile duct, we first analyzed the GSE32225 dataset which includes 104 CCA versus 6 normal bile duct samples. In addition, we analyzed the GSE32225 dataset which includes 149 CCA tissue samples versus 6 cases of benign biliary epithelia. For KAT2B gene expression analysis in cultured biliary epithelial and cancer cells from the GSE77984 dataset, the analysis includes 8 normal human biliary epithelial cell samples (NHC2#1–4, N_shC#1–4) and 6 CCA cell samples (EGI#2–4, E_pWPI#1–3). For KAT2B gene expression analysis in cultured biliary epithelial and cancer cells from the GSE144521 dataset, the analysis includes 6 normal human biliary epithelial cell samples (H69WCE_1–3, NHCWCE_1–3) and 6 CCA cell samples (EGIWCE_1–3, TFKWCE_1–3).

### CCA xenograft studies in SCID mice

The athymic nude NOD CB17-Prkdc/SCID mice were obtained from Jackson Laboratory (Bar Harbor, ME). For all animal studies, the procedures were carried out in strict accordance with the National Institutes of Health Guidelines for the Care and Use of Laboratory Animals. The handling of the mice and all experimental procedures were approved for this study by the Institutional Animal Care and Use Committee of Tulane University (Protocol #: 4159). To develop CCA xenografts with KAT2B overexpression, 1 × 10^6^ SG231 or HuCCT1 cells stably transfected with the pMSCV-KAT2B vector or control vector were inoculated into the left or right flank areas of the SCID mice. The animals were then monitored for tumor growth. After 7 weeks of inoculation, the mice were sacrificed and the tumors were collected.

### Statistics

Results are presented as mean ± standard error (SE) from a minimum of 3 replicates. Difference between groups was evaluated by SPSS 13.0 statistical software with one-way analysis of variance (ANOVA), Student’s t-test, and Log-rank test. Statistical graphs were plotted by GraphPad Prism 7.0 software and SigmaPlot statistical software. *P* value < 0.05 was considered as statistically significant.

## Results

### Downregulation of KAT2B mRNA in human CCA

**Fig. 1:**
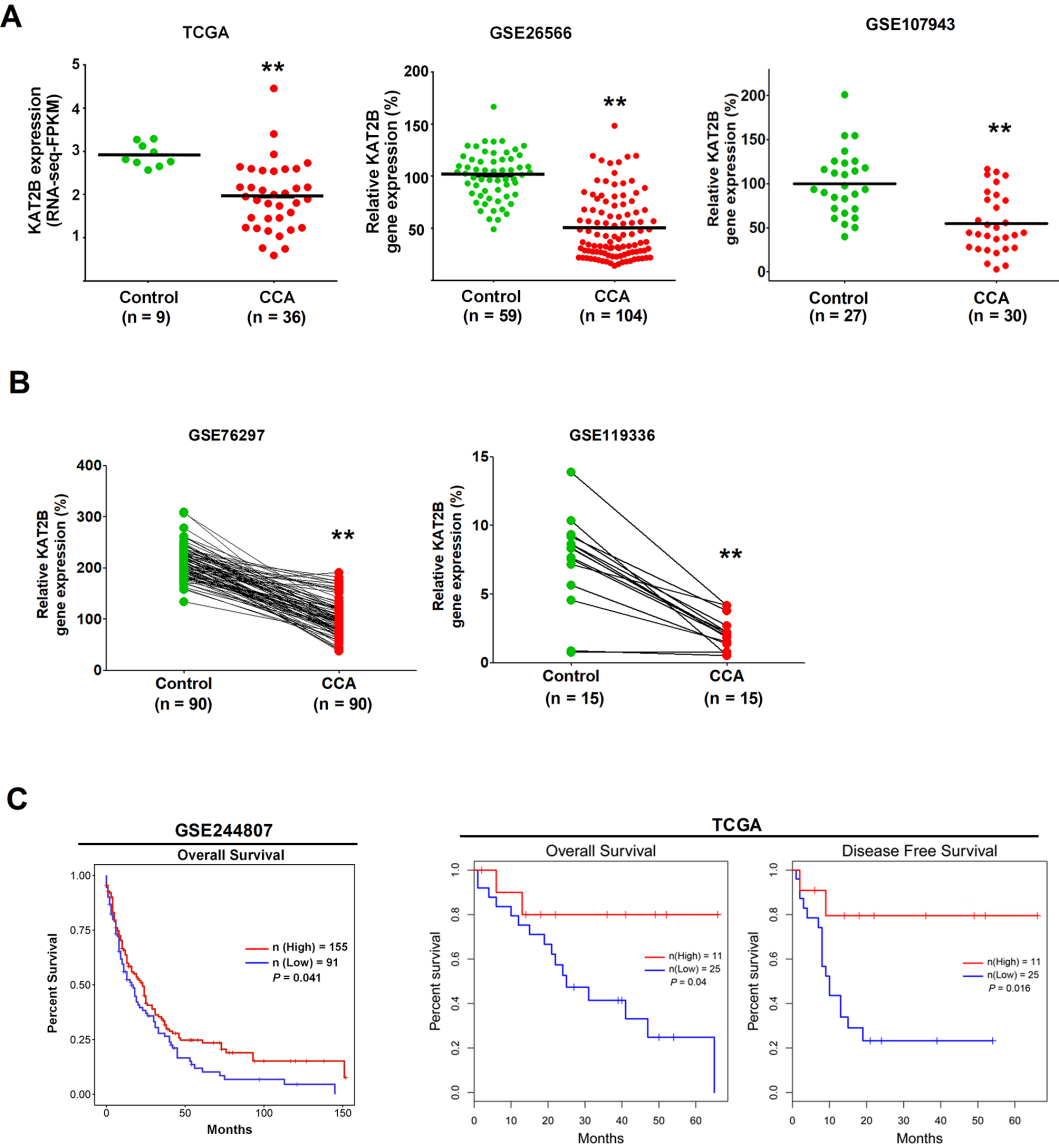
Informatics’ analyses of KAT2B expression in CCA tumor and its relationship with the prognosis of CCA patients. (**A**, **B**) Publicly available clinical cancer gene-expression data were downloaded from TCGA (The Cancer Genome Atlas Program) database and GEO (Gene Expression Omnibus) datasets to assess KAT2B expression in non-paired CCA cohort (**A**) and paired CCA cohort (**B**). (**C**) Kaplan-Meier survival curves for patients with CCA based on KAT2B expression from GSE244807 dataset and the TCGA database. **P < 0.01

We first analyzed the expression of KAT2B mRNA in human CCA tissues by using the TCGA database and the GEO datasets (GSE26566 and GSE107943). While TCGA RNA-Sequencing database contained the information from 36 CCA patients, the microarray dataset GSE26566 and the RNA-Sequencing dataset GSE107943 included 104 CCA samples and 30 CCA samples, respectively (these datasets contained non-paired tissue samples). Our analysis revealed downregulation of KAT2B mRNA in human CCA when compared with the non-cancerous liver tissues (Fig. 1A). In parallel, we analyzed the GEO datasets containing paired CCA and non-tumorous liver tissues including the microarray dataset GSE76297 (*n* = 90) and the RNA-Sequencing dataset GSE119336 (*n* = 15); this analysis also showed a low expression of KAT2B mRNA in human CCA tissues (Fig. 1B). Thus, analyses of both paired and non-paired human CCA tissue samples reveal that the expression of KAT2B mRNA is significantly downregulated in human CCA.

To further validate the downregulation of KAT2B in CCA, we performed additional analyses to compare the expression of KAT2B mRNA between human CCA tissues and non-cancerous bile ducts using the datasets containing BEC gene expression data (GSE26566, GSE32225). Analysis of the dataset GSE26566 revealed decreased KAT2B expression in CCA tissues (*n* = 104) in comparison to bile ducts (*n* = 6). However, analysis of the dataset GSE32225 revealed no significant difference in KAT2B expression between CCA tissues (*n* = 149) and non-cancerous biliary epithelia (*n* = 6) (Supplementary Fig. S1A). It is possible that the observed discrepancy may be related to the small numbers of bile duct samples available for the comparison. To help further clarify this, we analyzed the available datasets containing gene expression data from cultured human CCA cells and biliary epithelial cells (BECs) (GSE77984 and GSE144521); these analyses revealed downregulation of KAT2B in human CCA cells when compared to BECs (Supplementary Fig. S1B).

Finally, we analyzed the survival data from the datasets containing patient survival information (GSE244807 and TCGA-CHOL). While TCGA database contains information on both overall survival (OS) and disease-free survival (DFS), the GSE244807 dataset only provides OS data but not DSF data. Analyses of the GSE244807 survival data and the TCGA-CHOL survival data show that low expression of KAT2B mRNA is associated with poorer overall survival and disease-free survival of CCA patients (Fig. 1C).

### Shallow deletion of KAT2B gene contributes to decreased KAT2B mRNA expression in human CCA

**Fig. 2:**
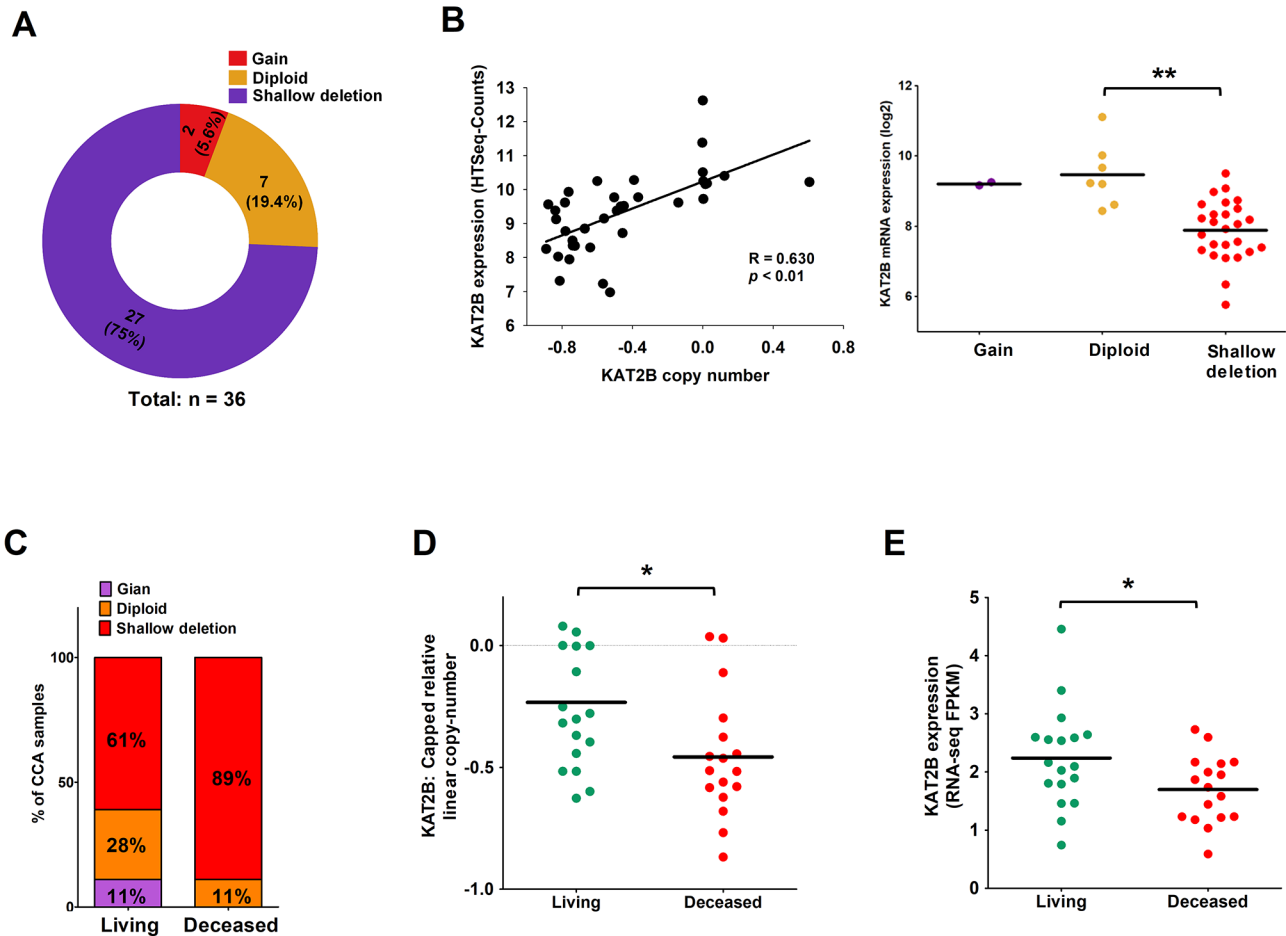
Shallow deletion of gene copy number contributes to the downregulation of KAT2B in CCA. (**A**) Analysis of the TCGA CCA dataset revealed that KAT2B gene copy number deletion was detected in 75% of CCA tumors (*n* = 27). (**B**) The co-relationship between KAT2B gene copy number and its mRNA expression from TCGA CCA cohort. (**C**, **D**) The CCA tissues from deceased patients exhibited higher proportion (89%) of shallow deletion (**C**) and lower level of KAT2B gene copy number (**D**). (**E**) The mRNA expression of KAT2B was lower in the CCA tissues from deceased patients than that from living patients. *P < 0.05, **P < 0.01

We next performed gene copy number variation analysis from TCGA database to determine whether gene copy number deletion might have contributed to the downregulation of KAT2B mRNA in human CCA. We observed that diploid and gain of KAT2B were found in only 19.4% (*n* = 7) and 5.6% (*n* = 2) of CCA samples, respectively. In contrast, shallow deletion of KAT2B was detected in 75% (*n* = 27) of CCA samples (Fig. 2A). There was no evidence of KAT2B amplification.

We then analyzed the correlation between KAT2B gene copy number and its mRNA level. Our data showed that the mRNA expression of KAT2B was positively correlated with KAT2B gene copy number in CCA tumors (Fig. 2B). The expression level of KAT2B was significantly decreased in CCA tumors with KAT2B gene shallow deletion (Fig. 2B). Notably, we found that the shallow deletion of KAT2B gene was associated with the survival status of CCA patients, as higher proportion of KAT2B gene shallow deletion was detected in the CCA tissues from deceased patients (89%) when compared with that from living patients (61%) (Fig. 2C). The CCA tissues from deceased patients showed a lower level of KAT2B gene copy number (Fig. 2D). Also, the expression of KAT2B mRNA was decreased in CCA tissues from deceased patients (Fig. 2E). Together, these findings indicate that loss of KAT2B gene copy number due to shallow deletion contributes to decreased KAT2B mRNA expression in human CCA.

### Forced overexpression of KAT2B inhibits human CCA cell growth, in vitro

**Fig. 3:**
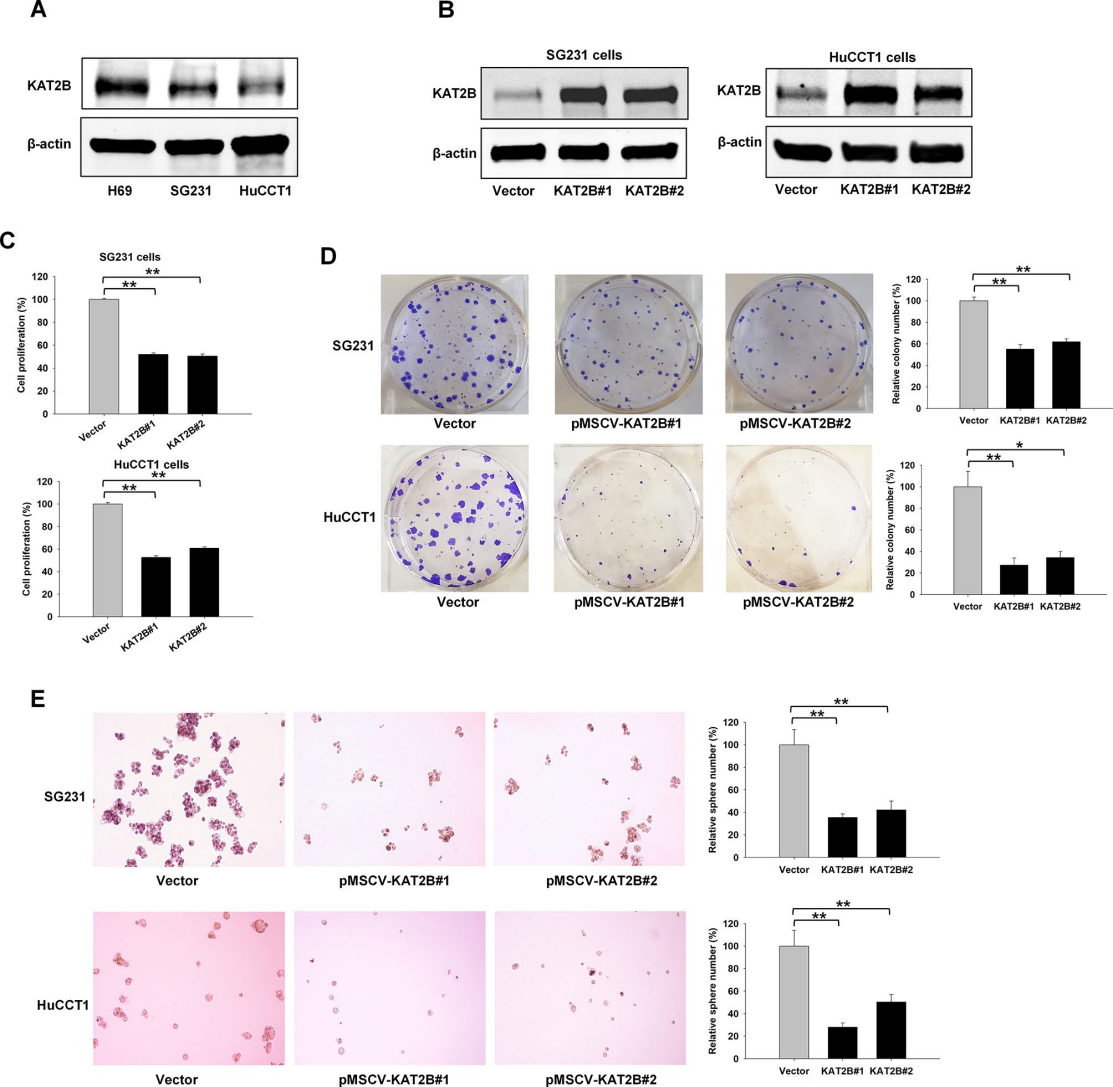
Forced overexpression of KAT2B inhibits CCA cell growth. (**A**) KAT2B protein in CCA cells (SG231 and HuCCT1) is lower than in noncancerous biliary epithelial cells (H69), as determined by Western blotting analysis. (**B**) SG231 and HuCCT1 cells were stably transfected with the pMSCV-KAT2B or the empty control vector. Two colonies for each cell line were selected after the cells were screened by puromycin. The level of KAT2B protein was analyzed by Western blotting assay. (**C**) Forced overexpression of KAT2B inhibited the cell proliferation of SG231 cells (*n* = 8) and HuCCT1 cells (*n* = 6), as measured by WST-1 assay. (**D**) Anchorage-dependent colony formation assay in SG231 and HuCCT1 cells with KAT2B overexpression (*n* = 6). (**E**) Anchorage-independent colony formation efficiency in SG231 cells (*n* = 6) and HuCCT1 cells (*n* = 6 for vector control and KAT2B#2, *n* = 5 for KAT2B#1) with KAT2B overexpression. **P < 0.01

To assess the functional impact of KAT2B in CCA, we examined the expression of KAT2B protein in human CCA cell lines (SG231 and HuCCT1) and in noncancerous human biliary epithelial cells (H69). Our data confirmed the low expression of KAT2B protein in CCA cells when compared to biliary epithelial cells (Fig. 3A). Next, we established human CCA cell lines (SG231 and HuCCT1) with stable overexpression of KAT2B using pMSCV-KAT2B vector. Satisfactory overexpression of KAT2B protein in CCA cells transfected with pMSCV-KAT2B vector was confirmed by Western blotting (Fig. 3B). WST1 cell proliferation assay showed that KAT2B overexpression significantly inhibited the proliferation of both SG231 and HuCCT1 cells (Fig. 3C). In contrast, knockdown of KAT2B expression in H69 cells by siRNA transfection significantly enhanced the proliferation of biliary epithelial cells (Supplementary Fig. S2A, B). We also assessed the effects of KAT2B overexpression on the colony formation ability of CCA cells. Our data showed that overexpression of KAT2B inhibited the anchorage-dependent as well as anchorage-independent colony formation capabilities of SG231 and HuCCT1 cells (Fig. 3D, E). These results indicate that stable overexpression of KAT2B inhibits human CCA cell growth, in vitro.

### Overexpression of KAT2B inhibits CCA growth, in vivo

**Fig. 4:**
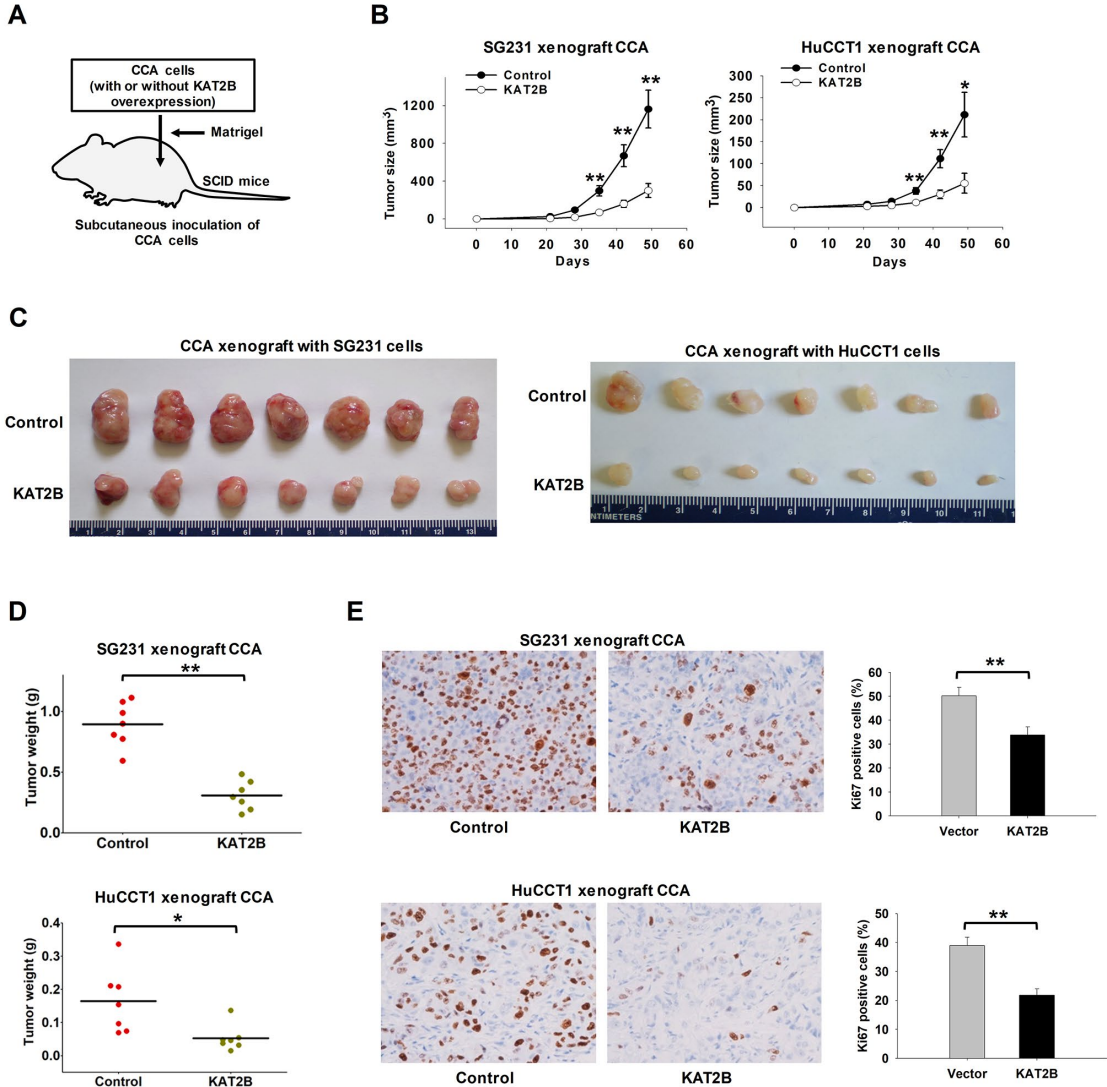
Overexpression of KAT2B suppressed the growth of CCA tumors in vivo. (**A**) Experimental outline for CCA xenograft in SCID mice. SG231 or HuCCT1 cells with or without KAT2B stable overexpression were mixed with Matrigel and then the cells were injected into the SCID mice subcutaneously. After 7 weeks of injection, the tumors were collected. (**B**) The growth curves of CCA tumors with or without KAT2B overexpression in SCID mice (*n* = 7). Tumor size was monitored every week. (**C**) Images of xenograft tumors recovered from 7 mice in each group. (**D**) The weight of CCA tumors with or without KAT2B overexpression in SCID mice (*n* = 7). (**E**) Immunohistochemistry for Ki67 using CCA xenograft tissues. The bar graphs show Ki67-positive cells in control and KAT2B-overexpressed tissues (*n* = 5). *P < 0.05, **P < 0.01

Following the above-described in vitro studies, we assessed the effect of KAT2B overexpression on CCA growth in SCID mice. For this purpose, SG231 cells or HuCCT1 cells with or without stable overexpression of KAT2B were injected subcutaneously into the flank of SCID mice and the animals were monitored for tumor growth (Fig. 4A). As shown in Fig. 4B and C, KAT2B overexpression in CCA cells significantly delayed the growth of CCA xenograft tumors. The CCA tumors with KAT2B overexpression exhibited significant decrease in tumor size and tumor weight when compared to the control tumors (Fig. 4B, D). By immunohistochemical analysis, we found that the tumor cells with KAT2B overexpression showed decreased staining for the cell proliferation marker Ki67 (Fig. 4E). These findings demonstrate that overexpression of KAT2B inhibited CCA growth in vivo.

### KAT2B induces the expression of the tumor suppressor NF2 in human CCA cells

**Fig. 5:**
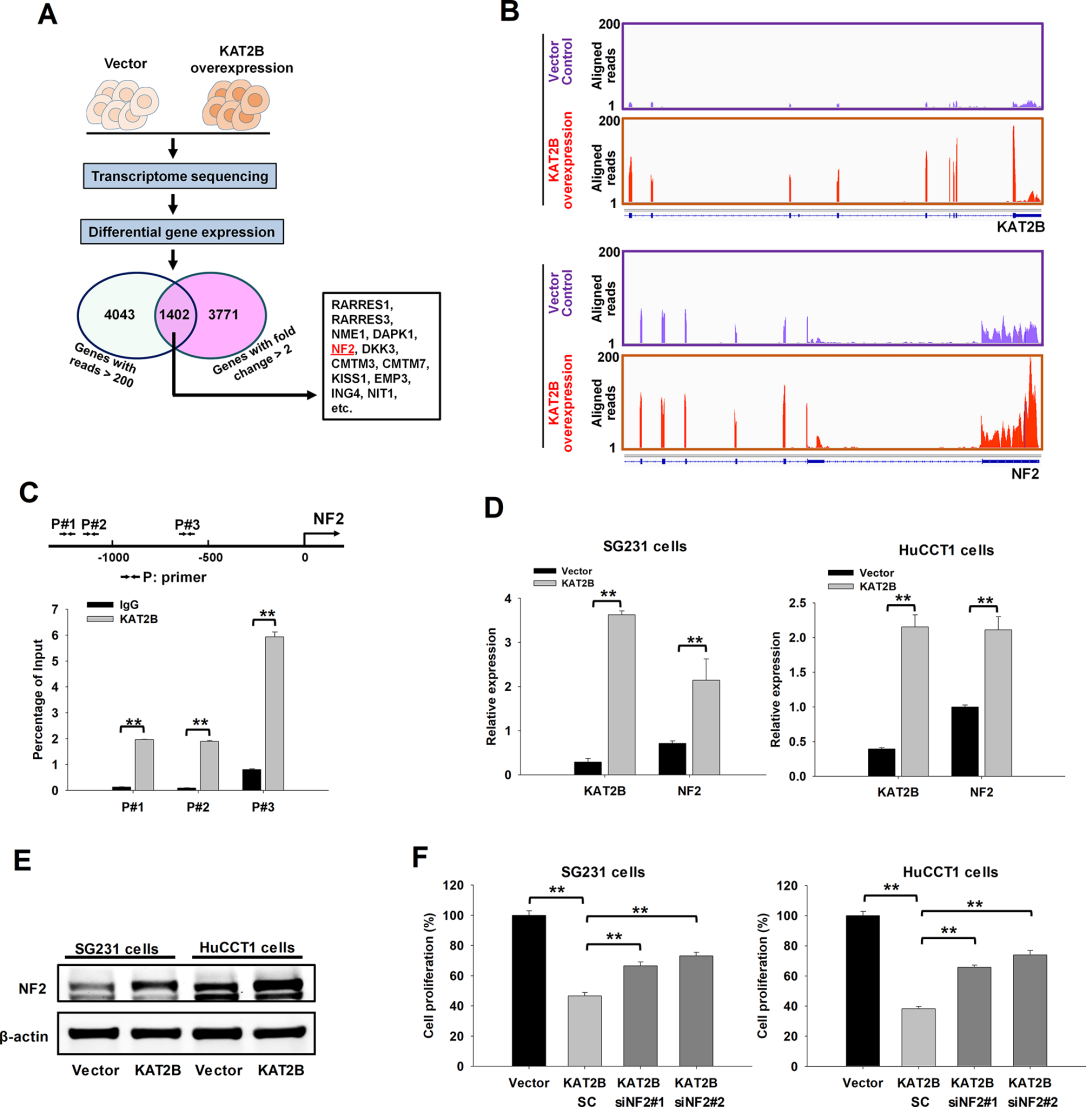
Upregulation of NF2 contributes to the tumor suppressing function of KAT2B. (**A**) RNA-Seq identified differentially expressed genes in KAT2B stably overexpressed cells when compared to the control cells. The transcriptome profiles of KAT2B stably overexpressed cells and control cells were analyzed by RNA-Seq and subjected to differential gene expression analysis. After filtering the low expression genes (with reads < 200), there were 1402 genes upregulated (> 2-fold) by KAT2B overexpression in HuCCT1 cells, which include various tumor suppressor genes. (**B**) Aligned RNA sequencing reads visualized through Integrative Genomics Viewer (IGV) presented the expression levels of KAT2B and NF2 in HuCCT1 cells. (**C**) ChIP-quantitative real-time PCR was used to analyze the enrichment of KAT2B in the promoter region of the NF2 gene (*n* = 8). Three primer pairs (P#1, #2 and 3) targeting the promoter region of NF2 were used for the assay. Rabbit immunoglobulin G (IgG) was used as negative control. (**D**) qRT-PCR confirmed that the mRNA levels of NF2 were upregulated by KAT2B overexpression in both SG231 cells and HuCCT1 cells (*n* = 4). (**E**) Western blotting assay showed that the protein levels of NF2 were upregulated by KAT2B overexpression in CCA cells. (**F**) WST-1 assay was used to evaluate the effects of NF2 siRNAs on the proliferation defect caused by KAT2B overexpression in SG231 and HuCCT1 cells (*n* = 8). **P < 0.01

To investigate the mechanism underlying KAT2B-mediated inhibition of CCA cell growth, we performed RNA-Seq analysis to detect transcriptomic changes in CCA cells with or without KAT2B stable overexpression. We reasoned that KAT2B might exert its tumor-suppressing function through upregulation of tumor-inhibitory genes in human CCA. We identified 1402 genes that were upregulated (> 2-fold) upon KAT2B overexpression in HuCCT1 cells (Fig. 5A). Functional enrichment analysis of the identified genes reveal that these genes are significantly enriched in cellular functions relevant to “’negative regulation of cell proliferation”, “positive regulation of apoptotic process"”, “cell adhesion”, “cell differentiation and “cell migration”; which are critically important for CCA tumor progression (**Supplementary Fig. S3**). Further analysis of the KAT2B-upregulated genes led to the identification of a number of tumor suppressor genes, including KISS1, RARRES1/3 and NF2, among others (Fig. 5A).

Among the identified genes, NF2 (Neurofibromatosis type 2) has been well-documented as an important tumor suppressor gene in biliary carcinogenesis [23–25]. In our study, we identified NF2 as one of the potential targets of KAT2B in human CCA. The RNA-Seq analysis revealed that NF2 mRNA expression was increased in HuCCT1 cells upon stable overexpression of KAT2B (Fig. 5B). Based on these findings, we carried out subsequent chromatin immunoprecipitation (ChIP) assay by using anti-KAT2B antibodies, and our data showed the enrichment of KAT2B in the promoter region of the NF2 gene (Fig. 5C). qRT-PCR analysis confirmed that NF2 mRNA expression was increased in both SG231 and HuCCT1 cells with overexpression of KAT2B (Fig. 5D). Accordingly, the level of NF2 protein was also significantly increased in CCA cells with KAT2B overexpression (Fig. 5E).

We next utilized siRNAs to knockdown NF2 expression in CCA cells with KAT2B stable overexpression. Our data showed that NF2 knockdown was able to rescue the reduction of cell proliferation caused by KAT2B overexpression (Fig. 5F).

Taken together, our experimental findings presented in the above sections reveal that KAT2B induces the expression of NF2 in CCA cells and that this regulatory mechanism is implicated in the regulation of CCA cell growth.

### KAT2B-induced NF2 regulates YAP activity in human CCA cells

**Fig. 6:**
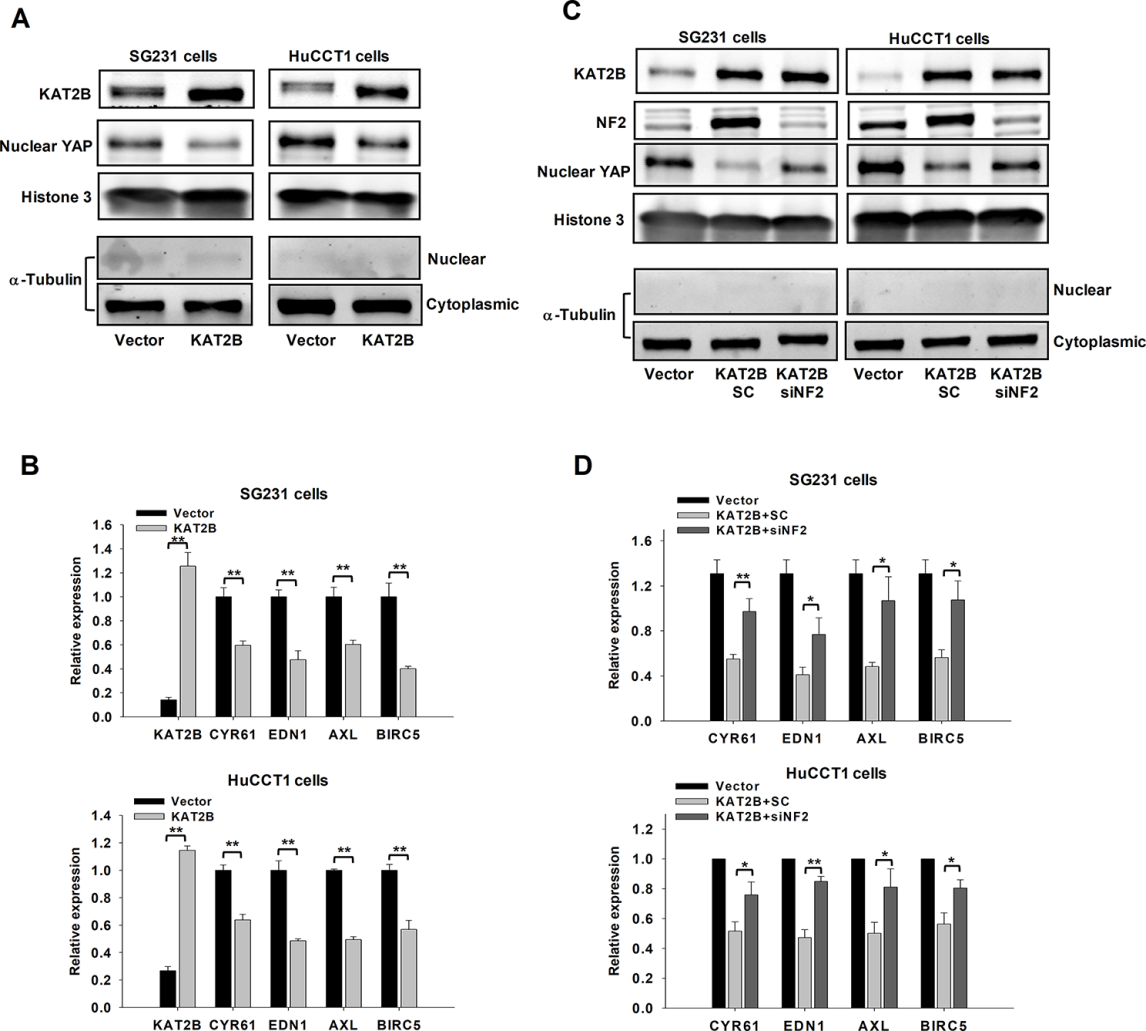
KAT2B overexpression decreases YAP activity via upregulation of NF2. (**A**) Western blot analysis to detect YAP nuclear accumulation. Nuclear proteins from SG231 and HuCCT1 cells were used for the analysis. Immunoblotting for histone 3 was used as control. Immunoblotting of α-Tubulin was used to exclude possible contamination of the cytoplasmic protein in the nuclear protein. (**B**) The mRNA levels of YAP target genes were analyzed by quantitative real-time PCR in SG231 and HuCCT1 cells with or without KAT2B overexpression (*n* = 4). (**C**) The effect of NF2 siRNA (NF2 siRNA#1 & siRNA#2) on KAT2B-induced downregulation of YAP protein was examined by Western blotting assay. (**D**) Quantitative real-time PCR was performed to assess the effects of NF2 siRNA (NF2 siRNA#1 & siRNA#2) on KAT2B-induced downregulation of YAP target genes (*n* = 5). *P < 0.05, **P < 0.01

NF2 has a well-defined role in the Hippo signaling pathway, where it regulates the membrane translocation and activity of LATS1/2, leading to inactivation of the Hippo pathway effector proteins (including YAP) [26–28]. As such, YAP is a major effector of NF2 which is implicated in the regulation of cell growth [29]. This is an important aspect in biliary carcinogenesis, as studies have revealed a negative correlation between NF2 and YAP in CCA [30]. Given that KAT2B induces the expression of NF2 as documented in the above sections, we performed further experiments to determine the status of YAP nuclear accumulation and its downstream target genes upon KAT2B overexpression in CCA cells. Our data showed that overexpression of KAT2B decreased the nuclear accumulation of YAP in both SG231 and HuCCT1 cells (Fig. 6A). We then analyzed the level of YAP in chromatin-bound fraction by extracting the insoluble chromatin fraction of CCA cells. The data showed that KAT2B overexpression decreased chromatin-bound YAP (Supplementary Fig. S4). Immunohistochemical staining further confirmed increased NF2 expression with decreased YAP expression in KAT2B overexpressed CCA xenograft models (Supplementary Fig. S5).

On the basis of the above experimental findings, we then assessed the effect of KAT2B overexpression on the expression of YAP downstream genes by qRT-PCR assay. Our data showed that overexpression of KAT2B significantly decreased the mRNA level of YAP downstream genes (including CYR61, EDN1, AXL, BIRC6) in CCA cells (Fig. 6B). Additionally, we analyzed the expression of YAP downstream genes using the RNA-seq data from HuCCT1 cells and identified a group of downregulated YAP target genes upon KAT2B overexpression (Supplementary Fig. S6). These findings indicated that KAT2B overexpression decreased the expression of YAP downstream genes in CCA cells.

We next performed rescue experiments to evaluate whether depletion of NF2 was able to reverse KAT2B-induced inhibition of YAP. To this end, KAT2B overexpressed CCA cells were transfected with NF2 siRNA and the cells were analyzed for the level of nuclear YAP and the expression of YAP downstream genes. We observed that siRNA depletion of NF2 was able to reverse the reduction of nuclear YAP caused by KAT2B overexpression in SG231 and HuCCT1 cells (Fig. 6C). Accordingly, our data showed that NF2 depletion partially restored the expression of YAP downstream genes in KAT2B-overexpressed CCA cells (Fig. 6D).

Taken together, our experimental findings presented above demonstrate an important role of KAT2B in the expression of NF2 and regulation of YAP activity in CCA cells.

### KAT2B interacts with SP1 to enhance NF2 gene transcription

While KAT2B is well recognized to function as a histone acetyltransferase and catalyzes histone H3 lysine 9 acetylation (H3K9ac), a modification associated with transcriptional activation [31, 32], there is also compelling evidence that KAT2B can regulate gene expression through mechanisms independent of its enzymatic action depending on specific cellular context [33–35]. Our data presented thus far demonstrate that KAT2B associates with promoter of the NF2 gene and regulates its expression in CCA cells. We then carried out further studies to delineate the mechanisms by which KAT2B regulates NF2 gene transcription. To determine whether the histone acetyltransferase activity of KAT2B is involved in the regulation of NF2 expression, we treated KAT2B stably overexpressed CCA cells with two different KAT2B histone acetyltransferase inhibitors, CPTH6 and L-Moses. Our data showed that inhibition of KAT2B acetyltransferase activity did not affect the expression of NF2 (Supplementary Fig. S7). These findings suggest that the regulation of NF2 by KAT2B is independent of the histone acetyltransferase activity of KAT2B.

**Fig. 7:**
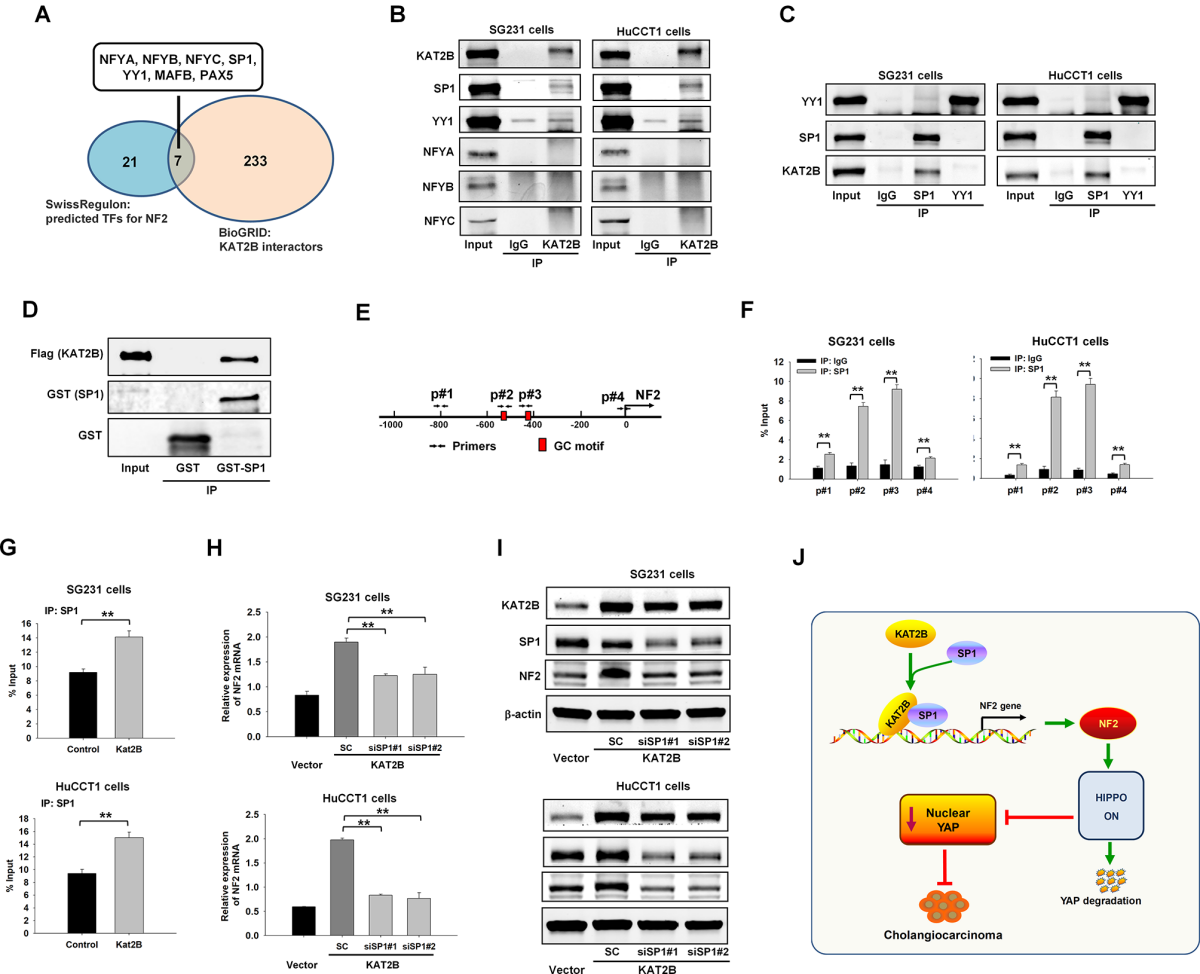
KAT2B regulates NF2 expression via recruiting SP1 to the promoter region of NF2 gene. (**A**) Venn diagram displays the overlapping genes of the potential transcription factors (TFs) for NF2 (from SwissRegulon) and KAT2B interactors (from BioGrid). (**B**, **C**) Protein immunoprecipitation was performed to validate the TFs interacted with KAT2B. B, the proteins were immunoprecipitated by KAT2B antibody; C, the proteins were immunoprecipitated by YY1 or SP1 antibody. IgG was used as negative control. (**D**) GST pull-down assay detecting the direct interaction between KAT2B and SP1. (**E**) GC-rich motif of NF2 promoter and ChIP-qPCR primer design. The GC-rich motifs at the promoter region of NF2 were predicted by Eukaryotic Promoter Database (cut-off p value: 0.001). Arrows with p#1–4 indicate the four primer sets that were used for the ChIP-qPCR analysis. Primer pairs #2 and #3 (p#2 and #3) were used to amplify the two GC motif regions; primer pairs #1 and #4 (p#1 and #4) were used to amplify non-GC motif regions. (**F**) ChIP-qPCR assay to show the enrichment of SP1 in the promoter region of NF2 (*n* = 4). (**G**) ChIP-quantitative real-time PCR analyses to examine the effect of KAT2B overexpression on the binding of SP1 to NF2 gene promoter (by using NF2 ChIP-qPCR primer pair 3 (p#3)) (*n* = 4). Rabbit immunoglobulin G was used as negative control. (**H**) The level of NF2 mRNA was analyzed by quantitative real-time PCR in vector control cells or KAT2B overexpressed cells with or without SP1 depletion (*n* = 4). (**I**) Western blotting for KAT2B, SP1 and NF2 in control cells or KAT2B overexpressed cells with or without SP1 depletion. (**J**) Schematic presentation highlighting the mechanisms of KAT2B in CCA. KAT2B inhibits CCA cell growth via interaction with SP1 to promote the expression of NF2 and thus activate Hippo pathway, which leads to subsequent inhibition of the oncogenic YAP signaling. **P < 0.01

Based on above results, we carried out further studies to identify the transcription factors (TFs) which may cooperate with KAT2B to regulate NF2 expression in CCA cells. By analyzing the putative TFs of NF2 predicted by SwissRegulon and KAT2B interactors predicted by BioGrid, we identified 7 transcription factors common in these two datasets, including NFYA, NFYB, NFYC, SP1, YY1, MAFB, PAX5 (Fig. 7A). Based on these findings, we performed subsequent immunoprecipitation studies to determine whether the above-mentioned TFs interact with KAT2B in CCA cells (MAFB and PAX5 were excluded because of their low expression [FPKM value = 0] in CCA cells based on our RNA-seq data). By using anti-KAT2B antibody for immunoprecipitation, we were able to detect the KAT2B-SP1 binding complex in CCA cells (Fig. 7B). For the detection of YY1, its interaction with KAT2B was questionable due to the presence of nonspecific band in the IgG control lanes (Fig. 7B). We then performed different sets of immunoprecipitation experiments using anti-SP1 antibody and anti-YY1 antibody. Our data showed that KAT2B was precipitated by anti-SP1 antibody, but not by anti-YY1 antibody (Fig. 7C). To examine the direct interaction between SP1 and KAT2B, we expressed and purified recombinant glutathione S-transferase (GST)–SP1 fusion protein from bacteria and performed GST pull-down assay using recombinant KAT2B-Flag protein. Our results confirmed the direct interaction between SP1 and KAT2B, as shown in Fig. 7D. Together, these findings demonstrate the interaction between KAT2B and SP1 in CCA cells.

SP1 is a zinc finger transcription factor which binds to GC-rich regions at the promoter of target genes to regulate the transcription of these genes [36, 37]. To further verify the role of SP1 in KAT2B-regulated NF2 expression, we searched for potential GC-rich motif at the promoter region of NF2 gene using the Eukaryotic Promoter Database (EPD). As shown in Fig. 7E, two potential GC-rich motifs were identified at the promoter of NF2 gene. Based on this information, we designed specific primer pairs (#1, #2, #3 and #4) targeting different portions of the NF2 gene promoter for ChIP-qPCR assay (primer pairs #2 and #3 amplify the two GC motif regions; primer pairs #1 and #4 amplify non-GC motif regions). Results of the ChIP-qPCR assays from both SG231 and HuCCT1 cells showed that SP1 was enriched in the promoter region of NF2 gene, especially in the GC motif region (Fig. 7F). This enrichment was significantly enhanced when the cells were transfected with the KAT2B expression vector (Fig. 7G). These findings support KAT2B interaction with SP1 in the NF2 gene promoter. We next performed further studies to determine whether knockdown of SP1 would attenuate KAT2B-induced NF2 gene expression. To this end, we transfected KAT2B overexpressed CCA cells with SP1 siRNA; the cells were then analyzed by qRT-PCR and Western blotting assays to detect the levels of NF2 mRNA and protein. We observed that KAT2B-induced upregulation of NF2 mRNA and protein expression was reduced when SP1 was knocked down (Fig. 7H, I). Together, our findings indicate that KAT2B is recruited by SP1 to the promoter of the NF2 gene which coordinately upregulate the expression of NF2 in CCA cells.

## Discussion

Deregulation of epigenetic regulators is importantly implicated in the development of various cancers including CCA [38]. Histone acetyltransferases (HATs) are known to have important effects on chromosome stability and gene transcription. Yet, the roles and underlying mechanisms of HATs in CCA development and progression are largely unknown. The current study provides the first evidence that the histone acetyltransferase KAT2B is an important tumor-suppressive molecule in CCA. Our experimental results show that KAT2B inhibits CCA cell growth in vitro and in mice. Detailed mechanistic studies reveal that KAT2B interacts with the transcription factor SP1 to induce the expression of the tumor suppressor gene NF2 in CCA cells, which leads to inhibition of oncogenic YAP signaling (Fig. 7J).

Downregulation of KAT2B has been reported in several cancers and is associated with poor prognosis, including hepatocellular carcinoma, esophageal squamous cell carcinomas, lung adenocarcinoma, gastric cancer, colorectal cancer and cervical carcinoma [39–44]. In hepatocellular carcinoma (HCC), studies have disclosed a tumor-suppressive role of KAT2B by inhibiting the growth/metastasis and accelerating the apoptosis of HCC cells [39, 45, 46]. However, the clinical significance of KAT2B expression in CCA has not been previously investigated. In this study, our analyses provide the first evidence that KAT2B expression is downregulated in human CCA. Importantly, patients with low KAT2B expression exhibited poorer prognosis when compared to patients with high KAT2B expression. Regarding the mechanisms that control the expression of epigenetic genes, studies have shown that the alterations of gene copy number contribute to the changes in the expression of epigenetic genes in various human cancers [47, 48]. As for KAT2B, Zhang and colleagues show that copy number deletion of KAT2B is present in multiple digestive cancers [49]. In human CCA, our analysis reveals that KAT2B is subjected to shallow deletion which leads to loss of gene copy number and thus decreased expression of KAT2B mRNA. These findings, along with the functional and mechanistic studies as detailed in the current paper, provide important evidence indicating an important tumor suppressive function of KAT2B in human CCA. In this context, it is possible that KAT2B expression may serve as a potential prognosis marker in CCA patients.

The mechanisms for KAT2B in regulating gene expression are complex. Although KAT2B is an important enzyme for acetylation of H3K9 to activate gene transcription [50, 51], it also regulates gene expression independently of its histone acetyltransferase activity [34, 35, 52]. In this study, our findings provide novel evidence implicating KAT2B in the upregulation of the tumor suppressor gene NF2 in CCA cells, which is independent of the histone acetyltransferase activity of KAT2B. Specifically, we show that KAT2B is recruited by SP1 to the promoter region of NF2 gene which leads to enhanced NF2 gene transcription.

As a Hippo pathway upstream gene involved in inactivation/destabilization of YAP/TAZ [25, 53], NF2 has been implicated in cholangiocarcinogenesis [23, 24]. For example, NF2 is downregulated in poorly differentiated CCA which is negatively correlated to YAP [30]. Inactivation of NF2 diminishes its ability to activate Hippo kinases, leading to activation of downstream YAP/TAZ and promoting cholangiocarcinogenesis [23–25]. Nonetheless, the regulatory mechanisms of NF2 in CCA remain to be further clarified. Our study identified KAT2B as an important regulator for NF2 in human CCA cells. This assertion is supported by the facts that (i) overexpression of KAT2B upregulates NF2 expression; (ii) KAT2B overexpression decreases the accumulation of nuclear YAP; (iii) KAT2B overexpression reduces the expression of YAP target genes; and (iv) KAT2B overexpression inhibits CCA cell growth in vitro and in mice. The role of NF2 in KAT2B-mediated effect is supported by the observation that NF2 depletion prevents KAT2B-induced inhibition of CCA cell growth.

While the current study is focused on the effect and mechanism of KAT2B in cholangiocarcinoma, it remains to be determined whether the KAT2B-NF2-YAP signaling pathway identified in CCA may also be applicable to other cancers. In this context, a previous study shows that KAT2B plays a tumor suppressive role in hepatocellular carcinoma (HCC) via regulating GLI1/BCL2 signaling pathway [45]. However, based on our RNA-seq data, the KAT2B/GLI1/BCL2 pathway is not applicable in cholangiocarcinoma. Thus, it is possible that some mechanisms of KAT2B may be cancer-specific or contexture-dependent. Further studies are warranted to investigate whether the KAT2B-NF2-YAP pathway identified in CCA may also operate in other cancer types.

In summary, the current study provides novel evidence that KAT2B plays an important tumor suppressive role in cholangiocarcinoma through interaction with SP1 to enhance the expression of NF2 which leads to subsequent inhibition of oncogenic YAP. Our findings disclose a novel KAT2B-NF2-YAP signaling axis in CCA which may be targeted for therapy.

### Electronic supplementary material

Below is the link to the electronic supplementary material.

Supplementary Material 1

Supplementary Material 2

## Data Availability

The data associated with this paper are available upon request to the corresponding author.

## Abbreviations

CCA: Cholangiocarcinoma
HATs: Histone acetyltransferases
H3K9ac: acetylated histone H3 lysine 9
KAT2B: K(Lysine) Acetyltransferase 2B
YAP: yes-associated protein
NF2: Neurofibromatosis type 2
SP1: Specificity Protein 1
TFS: transcription factors
