# Supplementary material for: The histone lysine acetyltransferase KAT2B inhibits cholangiocarcinoma growth: evidence for interaction with SP1 to regulate NF2-YAP signaling
Source: J Exp Clin Cancer Res. 2024 Apr 19;43:117. doi: 10.1186/s13046-024-03036-5 (PMC11027350; doi:10.1186/s13046-024-03036-5)
Supplement: Supplementary file 1 — Supplementary Material 1 [file 13046_2024_3036_MOESM1_ESM.docx]

**The histone lysine acetyltransferase KAT2B inhibits cholangiocarcinoma growth: Evidence for interaction with SP1 to regulate NF2-YAP signaling**

Wenbo Ma, Jinqiang Zhang, Weina Chen, Nianli Liu, Tong Wu

**Supplementary Figures and Figure Legends**

**Supplementary Figure S1.** (A) Analysis of KAT2B gene expression in CCA tissues versus normal bile duct samples by using the datasets GSE26566 and GSE32225. (B) Analysis of KAT2B gene expression in cultured CCA cells versus biliary epithelial cells (BECs) by using the datasets GSE77984 and GSE144521.

**Supplementary Figure S2.** The effect of KAT2B siRNA on H69 cell growth *in vitro*. H69 cells were transfected with two different KAT2B siRNAs (siKAT2B#1 and siKAT2B#2) or the scrambled control siRNA (SC). (A) Western blotting was performed to verify KAT2B depletion efficiency. (B) Knockdown of KAT2B by siRNA increases H69 cell growth, as measured by WST-1 cell proliferation assay (n = 10).

**Supplementary Figure S3.** GO (Gene Ontology) functional enrichment analysis of KAT2B-upregulated genes. The analysis was performed using Database for Annotation, Visualization and Integrated Discovery (DAVID).

**Supplementary Figure S4.** The effect of KAT2B overexpression on chromatin-bound YAP in CCA cells. SG231 and HuCCT1 cells were stably transfected with KAT2B expression plasmid and the insoluble chromatin fractions were obtained for Western blotting analysis.

**Supplementary Figure S5.** Immunohistochemical staining for KAT2B, NF2 and YAP in SG231 (A) or HuCCT1 (B) cell xenograft tumors with or without KAT2B overexpression.

**
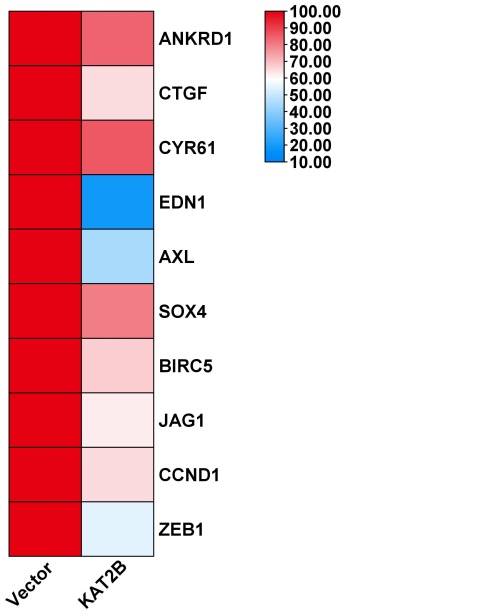
**

**Supplementary Figure S6.** Heatmap displaying YAP target genes reduced by KAT2B overexpression in HuCCT1 cells, as determined by RNA-Seq.

**Supplementary Figure S7.** The effects of KAT2B inhibitors on NF2 expression in KAT2B overexpressed CCA cells. The SG231 and HuCCT1 cells with stable KAT2B overexpression were treated with of KAT2B inhibitors CPTH6 (100 µM) and L-Moses (10 µM) for 48 h, and the cellular proteins were extracted for Western blotting analysis.
