# Supplementary material for: The histone lysine acetyltransferase KAT2B inhibits cholangiocarcinoma growth: evidence for interaction with SP1 to regulate NF2-YAP signaling
Source: J Exp Clin Cancer Res. 2024 Apr 19;43:117. doi: 10.1186/s13046-024-03036-5 (PMC11027350; doi:10.1186/s13046-024-03036-5)
Supplement: Supplementary file 2 — Supplementary Material 2 [file 13046_2024_3036_MOESM2_ESM.docx]

**Supplementary Table 1.** Primer sequences for qRT-PCR.

| Gene | Sense (5’ - 3’) | Antisense (5’ - 3’) |
| --- | --- | --- |
| KAT2B | AGGTTCCCCATGGATCTGAAAACC | AAAGACTCGCTGTAAGTCTGCCA |
| NF2 | ACAGAGCTGCTGCTTGGAGT | TGTTTCGGATTTCATTCCAC |
| AXL | TCAAGGTGGCTGTGAAGACGA | CGTTCAGAACCCTGGAAACAGAC |
| EDN1 | TGGGAAAAAGTGTATTTATCAGCA | TTTGACGCTGTTTCTCATGG |
| CYR61 | AGCCTCGCATCCTATACAACC | TTCTTTCACAAGGCGGCACTC |
| BIRC5 | GCCCAGTGTTTCTTCTGCTT | CCGGACGAATGCTTTTTATG |
| β-actin | CATGTACGTTGCTATCCAGGC | CTCCTTAATGTCACGCACGAT |

**Supplementary Table 2.** Primer sequences for ChIP-qPCR.

| Gene | Sense (5’ - 3’) | Antisense (5’ - 3’) |
| --- | --- | --- |
| NF2 P#1 | CGCCTGTAATCCTAGCACTT | TTTCTCCATGTTCGTCAGGC |
| NF2 P#2 | ATACAAAATTAGCCGGGCGT | GTTCAAGCGATTTTCCGGC |
| NF2 P#3 | GTTTCCCGGGTAAGGTCAGC | ACAGTGGCAGTCACTCTGAAT |
|  |  |  |
| NF2 p#1 | GAATATTCGCCGTGTGTCCG | TTCAAGTAGGCTCCGAGTGT |
| NF2 p#2 | GTGAAACTTTCCATTGGTGGAGTC | GGACTTTCCCGCTCCGTC |
| NF2 p#3 | CCTTCCCAGCCAATCGC | CAGCGCAACCGAACCG |
| NF2 p#4 | AGGCCTGTGCAGCAACT | CACGGCCTGCACTCTGA |

**Supplementary Table3.** siRNA sequences used in this study.

| siRNAs | Sequence |
| --- | --- |
| NF2  siRNA#1 | Sense: rArUrGrArGrCrUrUrCrArGrCrUrCrUrCrUrCrArArGrArGGA  Antisense: rUrCrCrUrCrUrUrGrArGrArGrArGrCrUrGrArArGrCrUrCrArUrGrC |
| NF2  siRNA#2 | Sense: rGrArCrArUrArCrCrArArGrCrUrUrCrArArCrCrUrCrArUTG  Antisense: rCrArArUrGrArGrGrUrUrGrArArGrCrUrUrGrGrUrArUrGrUrCrArG |
| SP1  siRNA#1 | Sense: rGrGrUrGrCrArArArCrCrArArCrArGrArUrUrArUrCrArCAA  Antisense: rUrUrGrUrGrArUrArArUrCrUrGrUrUrGrGrUrUrUrGrCrArCrCrUrG |
| SP1  siRNA#2 | Sense: rGrGrUrGrArGrArUrArGrUrArArArArCrArCrUrUrArUrUCC  Antisense: rGrGrArArUrArArGrUrGrUrUrUrUrArCrUrArUrCrUrCrArCrCrArC |
| KAT2B  siRNA#1 | Sense: rGrCrArUrCrUrArGrGrUrUrUrUrUrGrArUrArArUrUrCrUGT  Antisense: rArCrArGrArArUrUrArUrCrArArArArArCrCrUrArGrArUrGrCrUrU |
| KAT2B  siRNA#2 | Sense: rCrGrArUrCrUrCrCrCrArArUrGrArUrGrArUrArUrUrUrCTG  Antisense: rCrArGrArArArUrArUrCrArUrCrArUrUrGrGrGrArGrArUrCrGrCrA |
